# Intra-abdominal Versus Descending Thoracic Cannulation Techniques for Abdominal Normothermic Regional Perfusion

**DOI:** 10.1097/TXD.0000000000002006

**Published:** 2026-09-09

**Authors:** Grace Boyne, Olivia Claire Walker, Jinyu Du, Johanna Bayer, Juan Sebastian Danobeitia, Geoffrey Funk, Amar Gupta, Seung-Hee Lee, Eric Martinez, Kara Monday, Scott Noesges, Murphy Rayle, Giuliano Testa, Anji Wall

**Affiliations:** 1 Regional Perfusion Services LLC, Richardson, TX.; 2 Division of Abdominal Transplantation, Department of Surgery, Baylor University Medical Center, Baylor Scott and White Health, Dallas, TX.; 3 Southwest Transplant Alliance, Dallas, TX.

## Abstract

**Background.:**

Abdominal normothermic regional perfusion (A-NRP) is postmortem perfusion of organs in the abdominal cavity and can be performed using multiple approaches, including femoral, intra-abdominal, and thoracic. Our program began with surgeons performing intra-abdominal cannulations with mechanical or balloon occlusion of the descending thoracic aorta for A-NRP procedures. Recently, we developed a novel technique using the descending thoracic aorta for arterial cannulation with right atrial venous drainage.

**Methods.:**

All A-NRP cases performed by Regional Perfusion Services from July 1, 2024, to June 30, 2025, were retrospectively reviewed. Intraoperative parameters were collected for comparison.

**Results.:**

During the study period, 28 intra-abdominal normothermic regional perfusion (IA-NRP) cannulations and 47 descending thoracic normothermic regional perfusion (DT-NRP) cannulations were performed by 16 surgeons. Median cannulation time was shorter in the DT-NRP cohort (8.5 versus 10.0 min; *P* = 0.016). Median time on NRP was shorter in the IA-NRP cohort (61.5 versus 91.0 min; *P* = 0.006). The median decrease in lactate was larger in the DT-NRP cohort (2.9 versus 2.3 mmol/dL; *P* = 0.024). The median number of transfusions used was higher in the IA-NRP cohort (3.0 versus 0.0; *P* < 0.001). The learning curves show a quicker descent in time and a lower nadir for the DT-NRP cases, indicating this technique may have a quicker learning curve and be a more efficient approach to cannulation.

**Conclusions.:**

DT-NRP cannulation can be implemented for A-NRP with a quick learning curve, providing an additional cannulation option to the IA-NRP approach.

## Graphical abstract

**Figure:**
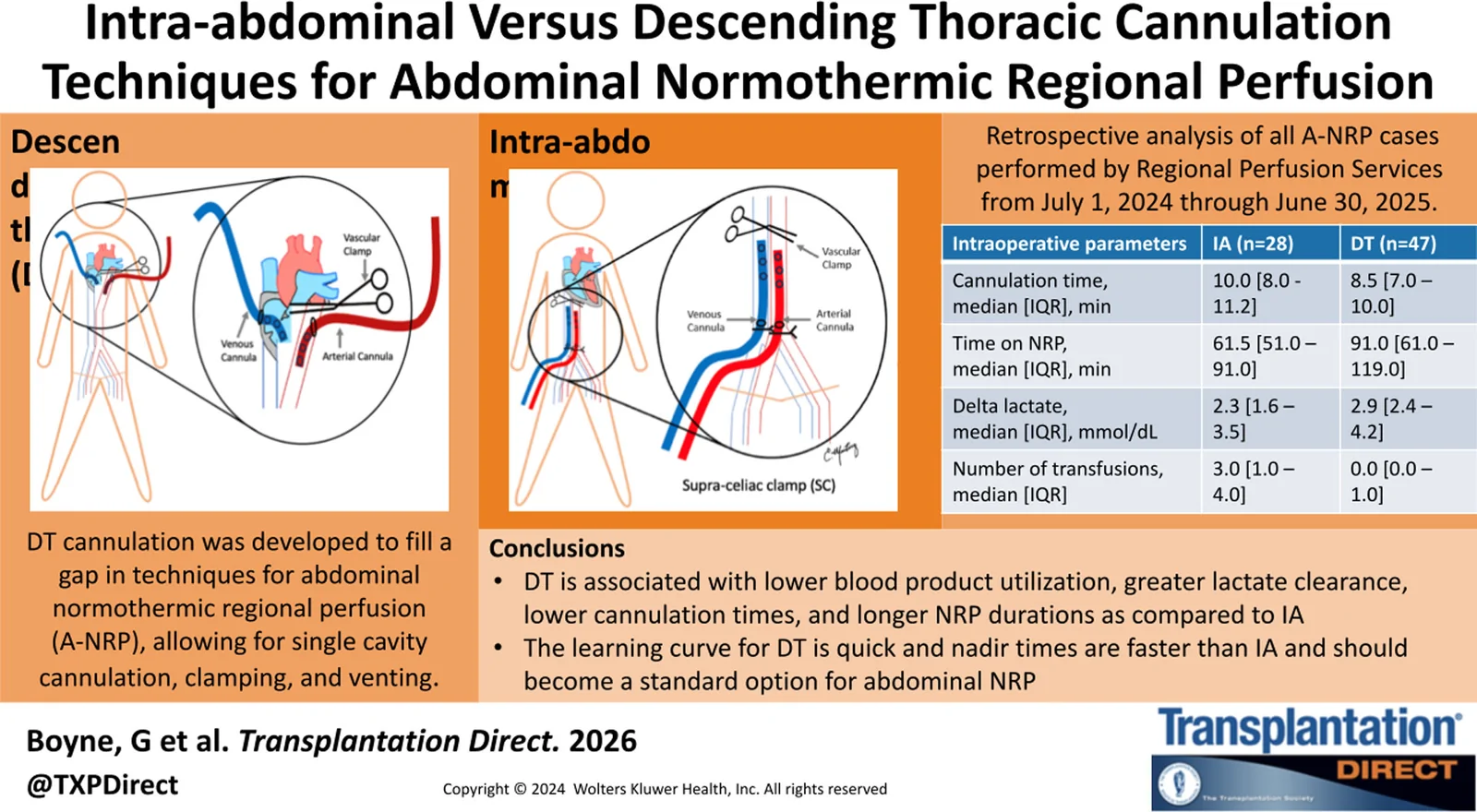

## INTRODUCTION

Normothermic regional perfusion (NRP) has become an accepted and often preferred technique for the recovery of organs following donation after circulatory death (DCD) in many countries.^1-4^ The goal of NRP is to provide blood-based oxygenated perfusion to the areas of the body where the organs intended for transplantation are located. There are 2 types of NRP: abdominal (A-NRP), which perfuses only abdominal organs, and thoracoabdominal NRP, which perfuses thoracic and abdominal organs. Both types of NRP can be achieved with multiple cannulation strategies. Throughout the world, different preferences for cannulation have been developed based on surgeon expertise and training, national policies governing organ donation, and organ recovery logistics. For example, NRP program development in the United States was led by cardiothoracic teams who performed thoracoabdominal NRP with central cannulation.^5^ Abdominal teams trained by cardiothoracic surgeons in the United States often adopted a central cannulation technique.^6^ In contrast, NRP in Spain started in an uncontrolled setting using percutaneous femoral cannulation, which was translated into premortem cannulation in the controlled setting.^2,7^ Each cannulation strategy has benefits and limitations, and there may be times at which the preferred cannulation strategy cannot be used because of donor anatomy, past surgical history, donor logistics, or donor hospital policies.^8^

For A-NRP, 2 techniques have been described: femoral cannulation and intra-abdominal cannulation (IA-NRP). Femoral cannulation is most often performed in the premortem period and involves cannulation of the femoral artery and vein, as well as cannulation of the contralateral femoral artery for the introduction of an intra-aortic occlusion balloon to limit perfusion to the abdominal cavity. Its limitations are that the venting above the balloon still requires opening the chest, so NRP cannot be started outside of an operating room, even if cannulation is performed in an intensive care unit or postanesthesia care unit. IA-NRP cannulation avoids the need for premortem interventions but is challenging in patients who have had previous abdominal operations, are morbidly obese, or have distal aortic atherosclerosis. In addition, it generally requires opening both the abdomen for cannulation and the chest to clamp and vent, making the cannulation process longer than other approaches.

Given challenges and limitations with the 2 most common A-NRP techniques, our team developed a new approach to A-NRP, termed descending thoracic aortic cannulation (DT-NRP). In this approach, the descending thoracic aorta is cannulated and vented, and the venous return cannula is placed through the right atrial appendage. This approach does not require premortem interventions and is performed in a single cavity, allowing for greater efficiency in cannulation. It is also not limited by morbid obesity or prior abdominal operations. DT-NRP is not indicated in a redo chest and can be challenging in patients who have scarring in the left chest from infection or instrumentation. Moreover, the thoracic cannulation technique eliminates the need to clamp or dissect individual head vessels, expedites the initiation of NRP, reduces operative complexity, and provides alternative options based on the disease process of the descending aorta. With the increased age of DCD donors, the comorbidities and disease processes can create surgical complications leading to dissected aortas and/or renal arteries, rendering the organ untransplantable. The physiology of this approach may also facilitate superior venous drainage from both the superior and inferior vena cava (IVC), potentially improving NRP circuit dynamics and limiting transfusion requirements, leading to better abdominal organ perfusion.

Having recognized the clinical value of adding the DT-NRP procedure to our practice, we elected to compare it to our intra-abdominal approach. This study aimed to compare procedural efficiency and efficacy between DT-NRP and IA-NRP cannulation techniques. Specifically, we compared intraoperative parameters, including cannulation time, time on NRP, blood product utilization, and lactate clearance, between DT-NRP and IA-NRP approaches. In addition, we assessed the learning curve associated with cannulation efficiency over time.

## MATERIALS AND METHODS

### Study Design

This study is a retrospective analysis of all A-NRP cases performed by Regional Perfusion Services from July 1, 2024, to June 30, 2025. Intraoperative parameters, including total and functional warm ischemia times, cannulation times, NRP durations, flows, transfusions, and lactate clearance, were collected. Flow dynamics, including highest flow, lowest flow, and calculated target flow, were analyzed. To account for differences in NRP duration between groups, an exploratory duration-normalized lactate range ([highest lactate–lowest lactate]/NRP duration) was calculated. Lactate measurements were not time-ordered; this metric reflects the magnitude of lactate change relative to NRP duration rather than a true lactate clearance rate or longitudinal slope. Target flow was calculated as 70% of body surface area multiplied by a cardiac index of 3.0 L/min/m^2^. Ratios of observed to target flow were used to assess perfusion adequacy. In addition, cannulation time over case number was compared between groups. A Wilcoxon signed-rank test was conducted on discrete and continuous variables, boxplots were used to present key variables, and a learning curve was made to compare cannulation time over chronological case order. This study was approved by the Baylor University Medical Center institutional review board (No. 025-529). The requirement for informed consent was waived because the study involved a retrospective analysis of data from deceased donors. This study was conducted in accordance with both the Declarations of Helsinki and Istanbul. This study adheres to Strengthening the Reporting of Observational Studies in Epidemiology guidelines (see STROBE checklist, **Table S1** [**SDC**, https://links.lww.com/TXD/A902]).^9,10^

### Surgical Technique

The DT-NRP cannulation approach begins with a rapid median sternotomy and placement of a chest retractor. The pericardium is excised to expose the heart. Venous drainage is established via the right atrium using a 29/29/29F (Edwards TF292902). The cannula is inserted through an atriotomy, directed to the IVC, and secured to the atrial wall with a Babcock clamp, Rommel tourniquet, or endoloop, based on surgeon preference. Following venous cannulation, the left pleural space is opened, and the left lung is retracted medially to expose the descending thoracic aorta. The aorta is encircled with umbilical tape for securing the cannula after placement. An aortic cross-clamp is placed on the descending thoracic aorta distal to the left subclavian artery and proximal to the planned cannulation site to prevent cerebral and thoracic perfusion. An 18F Medtronic EOPA (Medtronic 77418) or 20F Medtronic EOPA (Medtronic 77420) arterial cannula is inserted antegrade into the descending thoracic aorta through an aortotomy, secured in place with a Babcock clamp, and reinforced with the umbilical tape. The aorta is vented proximal to the clamp with a 20F (Medtronic 12112) drop sucker to collect volume back to the circuit. The aortic bifurcation in the abdomen is not clamped while on NRP in this technique.

The IA-NRP cannulation approach begins with a laparotomy and exposure of the aortic bifurcation and the adjacent IVC. Both vessels are encircled with umbilical tape. The distal aorta is clamped with a Kelley or other heavy clamp, and an anterior aortotomy is made. If a Coda balloon catheter (Cook Medical) is being used, it is introduced into the aortotomy first and inflated, wedge-tested, and secured to the skin with a hemostat. Next, an 18F Medtronic EOPA (Medtronic 77418) or 20F Medtronic EOPA (Medtronic 77420) arterial cannula is inserted into the aorta and secured with a Babcock clamp. The arterial cannula is connected to the arterial inflow tubing of the pump with a wet-to-wet connection. A venotomy in the IVC just cranial to the iliac confluence is made, and a 29/29/29F (Edwards TF292902) venous cannula is inserted cranially with the tip terminating at the level of the hepatic veins.

## RESULTS

**FIGURE 1.**
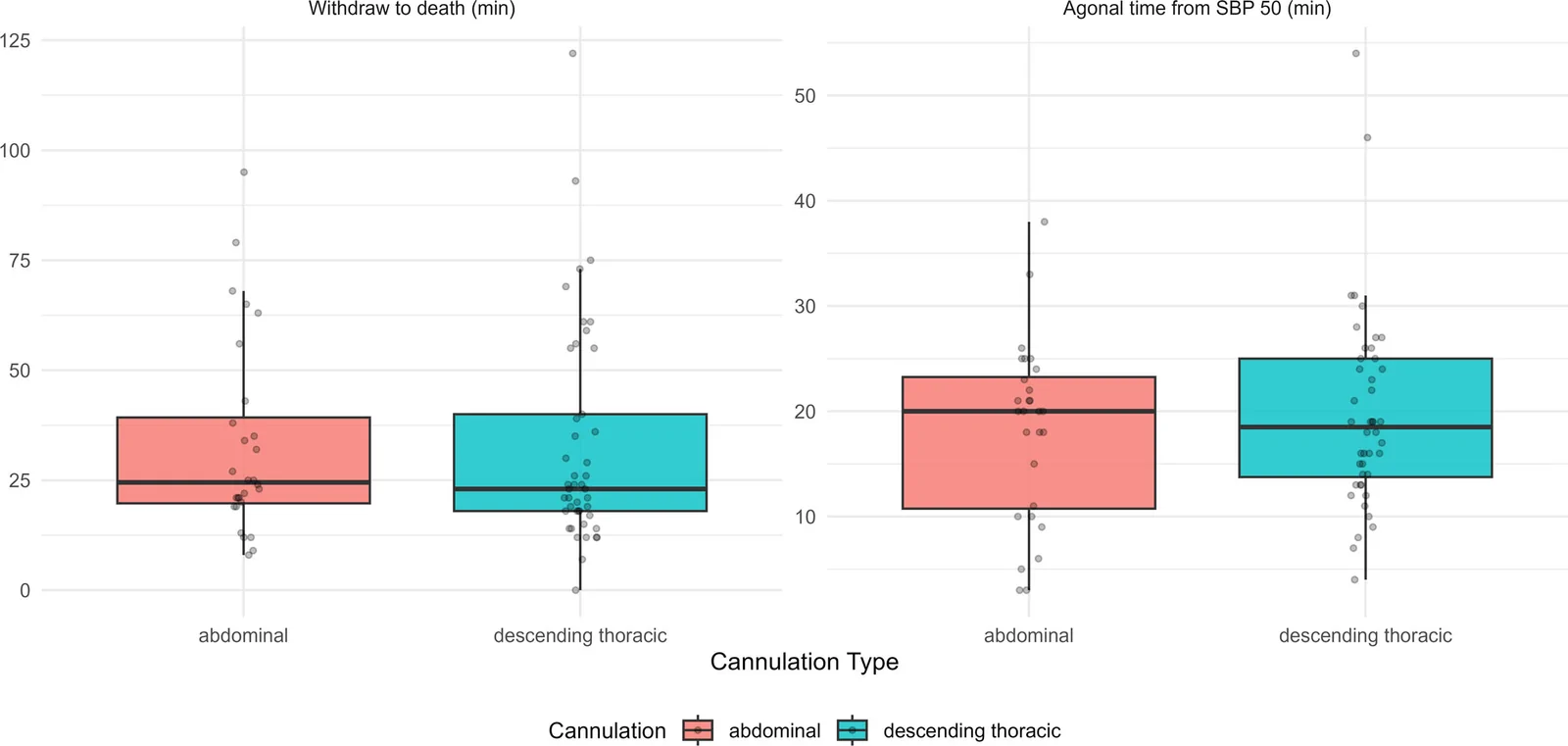
Withdrawal-to-death time and agonal time from SBP 50 by cannulation type. SBP, systolic blood pressure.

During the study period, 75 A-NRP procedures were performed, with 28 cases using IA-NRP and 47 cases using DT-NRP. Across these cases, a total of 16 cannulating surgeons performed at least 1 procedure, and 5 perfusionists were involved in at least 1 procedure. No significant differences were observed between groups in median (interquartile range [IQR]) time from withdrawal to declaration of death (24.5 [19.8–39.2] min IA-NRP versus 23.0 [18.0–40.0] min DT-NRP; *P* = 0.625) or agonal time from systolic blood pressure <50 mm Hg to initiation of NRP (20.0 min [10.8–23.2 min] IA-NRP versus 18.5 min [13.8–25.0 min] DT-NRP; *P* = 0.808). Figure 1 shows box plot comparisons of withdrawal to death time and agonal time.

**FIGURE 2.**
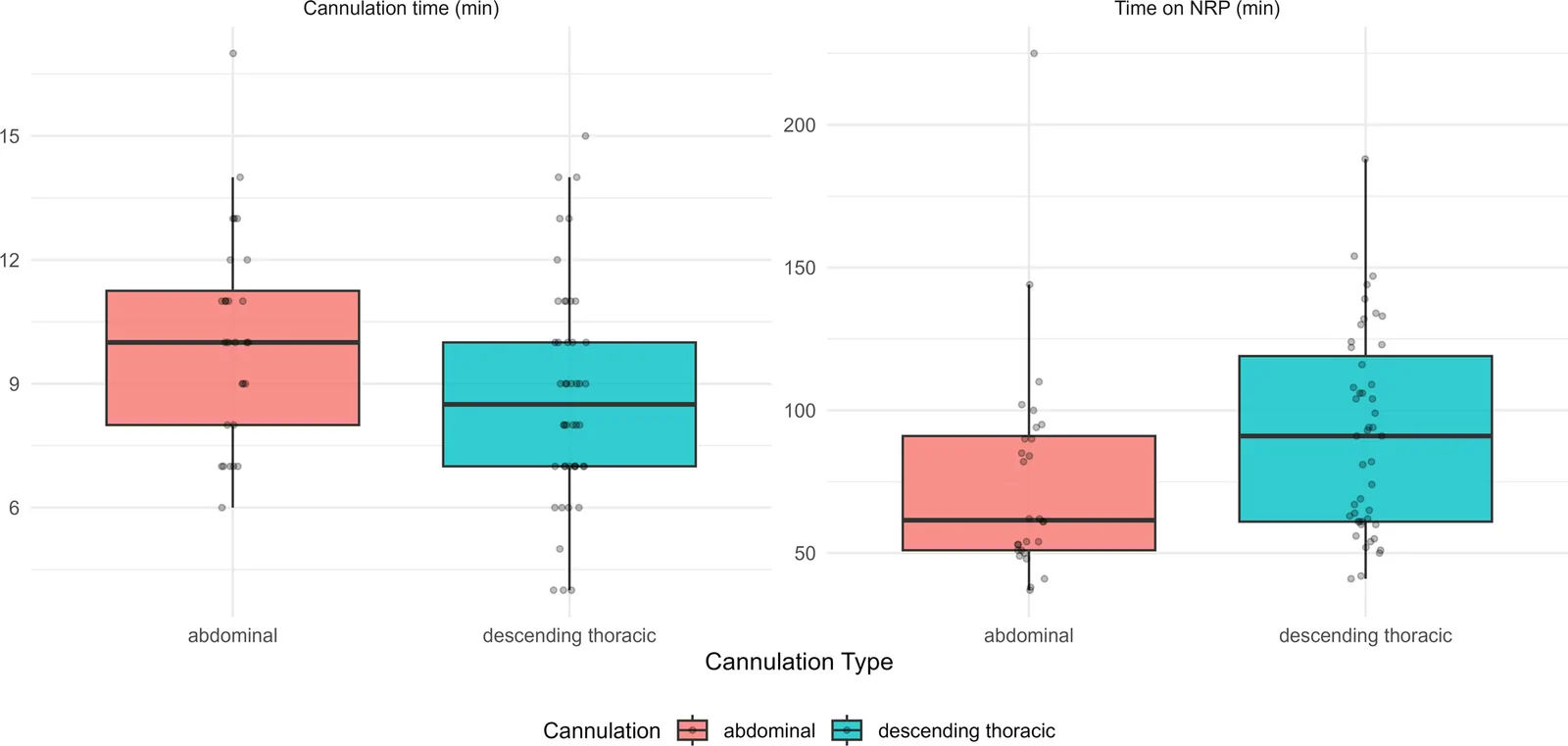
Cannulation time and NRP duration by cannulation type. NRP, normothermic regional perfusion.

The DT-NRP group demonstrated significantly shorter median [IQR] cannulation times compared with the IA-NRP group (10.0 min [8.0–11.2 min] IA-NRP versus 8.5 min [7.0–10.0 min] DT-NRP; *P* = 0.016). DT-NRP cases had longer median (IQR) NRP durations than IA-NRP (61.5 min [51.0–91.0 min] IA-NRP versus 91.0 min [61.0–119.0 min] DT-NRP; *P* = 0.006). Figure 2 shows box plot comparisons of cannulation times and NRP duration.

**FIGURE 3.**
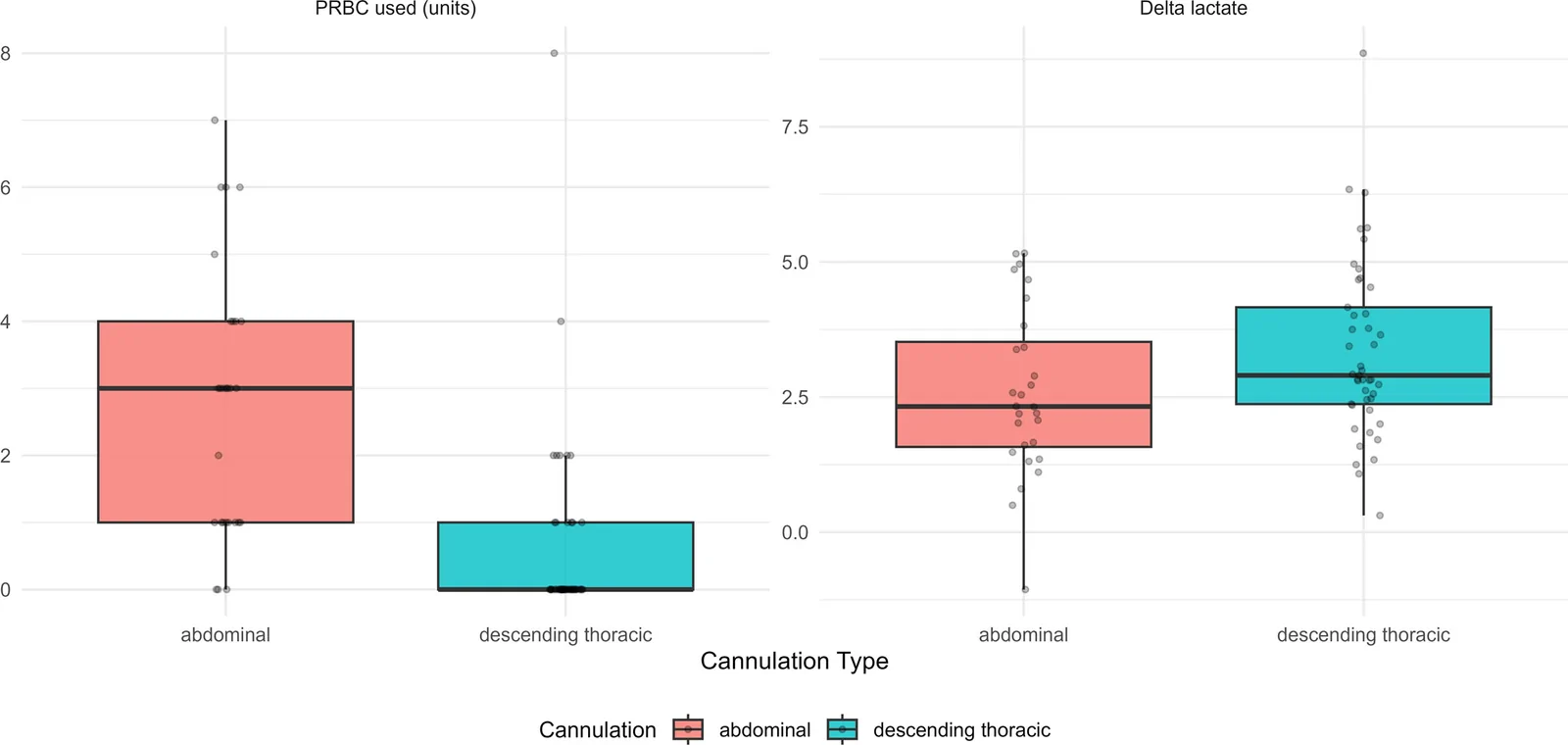
PRBCs used and delta lactate by cannulation type. PRBCs, packed red blood cells.

**FIGURE 4.**
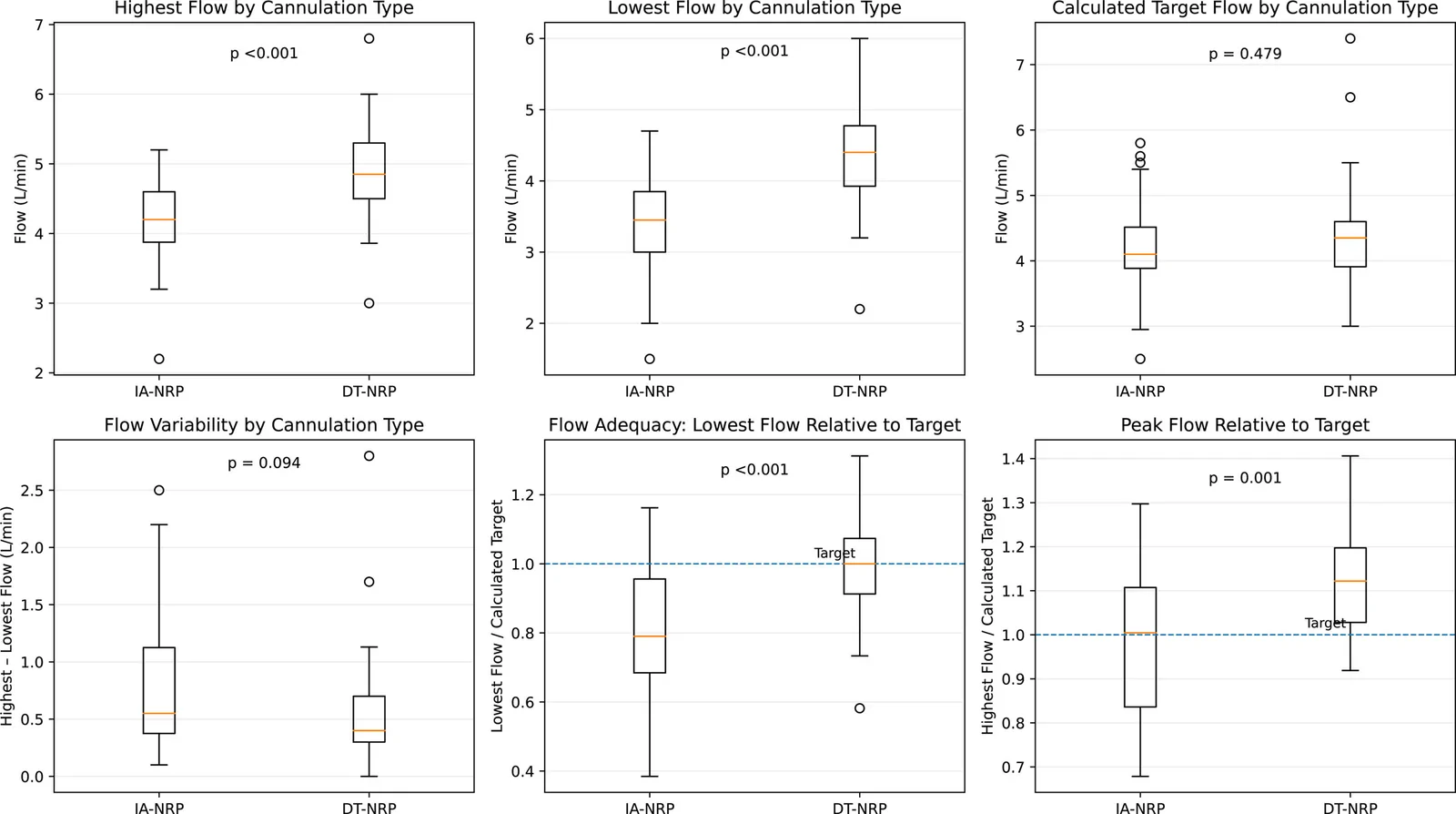
Comparison of flow dynamics by cannulation type. DT-NRP, descending thoracic normothermic regional perfusion; IA-NRP, intra-abdominal normothermic regional perfusion.

Median (IQR) packed red blood cell (PRBC) transfusion was significantly lower in the DT-NRP group compared with the IA-NRP group (3.0 units [1.0–4.0 units] IA-NRP versus 0.0 units [0.0–1.0 units] DT-NRP; *P* < 0.001). Median (IQR) delta lactate, defined as the change in lactate from NRP initiation to conclusion, was significantly higher in the DT-NRP group (2.3 mmol/L [1.6–3.5 mmol/L] IA-NRP versus 2.9 mmol/L [2.4–4.2 mmol/L] DT-NRP; *P* = 0.024). Figure 3 shows box plot comparisons of PRBCs used and delta lactate. Duration-normalized median lactate range was comparable in both groups (0.0339 lactate/min IA-NRP versus 0.0342 lactate/min DT-NRP; *P* = 0.8430). DT-NRP achieved significantly higher peak flow rates and trough flow rates compared with IA-NRP (both *P* < 0.001), despite similar calculated target flows (*P* = 0.479). DT-NRP also more consistently met or exceeded target flow requirements, as reflected by flow adequacy: lowest flow relative to target (*P* < 0.001) and peak flow to target ratios (*P* = 0.001). Figure 4 shows box plot comparisons of flow dynamics.

Rapid recovery procurement was required for 16 IA-NRP cases, including 4 heart, 10 lung, and 2 combined heart and lung recoveries. Heart recoveries had the longest median NRP time (73.5 min) and the highest mean PRBC utilization (4.75 units), followed by combined heart-lung recoveries (71.5 min, 4.5 units). Four heart procurements used the Organ Care System, requiring blood removal for pump priming, which likely contributed to the higher PRBC utilization observed in heart and heart-lung recoveries compared with other IA procurements. Lung recoveries had a median NRP time of 58 min and a mean PRBC utilization of 3.4 units, including 2 cases combining lung rapid recovery in the DT-NRP cohort. Other IA procurements had a similar median NRP time (57.5 min) but lower mean PRBC utilization (1.25 units). Additionally, 2 lung rapid recoveries occurred in the DT-NRP cohort.

**FIGURE 5.**
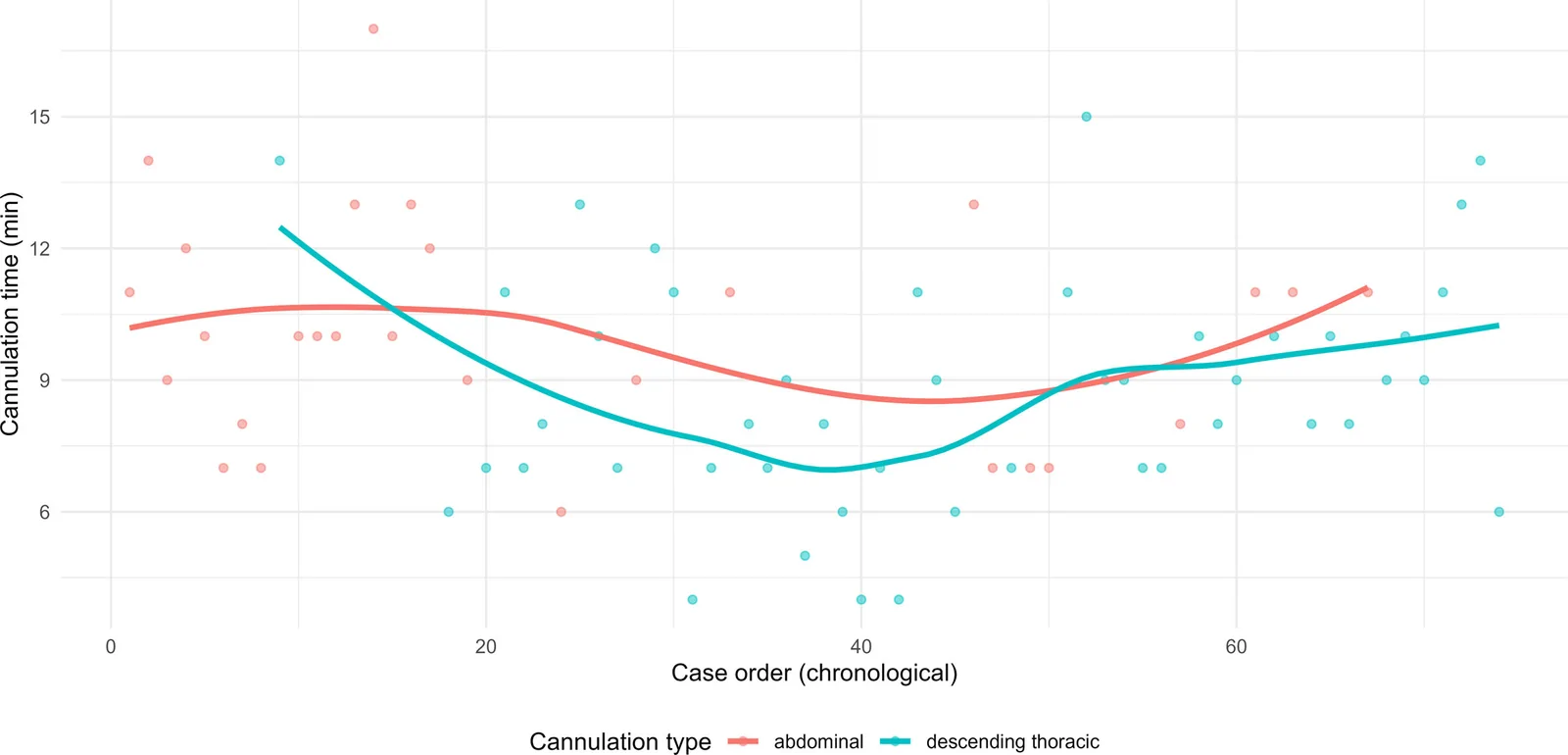
Learning curve of cannulation time for intra-abdominal and descending thoracic normothermic regional perfusion (NRP).

Figure 5 shows a comparison of the cannulation times versus case number for IA-NRP versus DT-NRP cannulations. The DT-NRP cannulation time slope showed a more rapid decline and lower nadir than the IA-NRP slope, with both uptrending after 50 cases.

## DISCUSSION

The current study provides a retrospective comparison of 2 techniques for A-NRP: DT-NRP and IA-NRP. Overall, we found that the DT-NRP technique was a safe, effective, and efficient approach to A-NRP and was noninferior to the IA-NRP approach. While there was no difference in time to death or functional warm ischemic times between groups, there were differences in cannulation times, transfusions, pump flows, lactate trends, and NRP duration.

The current study found that the DT-NRP approach was associated with shorter cannulation times and longer NRP duration than the IA-NRP approach. The shorter cannulation times are likely because of several factors. First, DT-NRP is performed in a single cavity versus IA-NRP, which generally requires access to both the abdomen and chest. Second, the descending thoracic aorta is more easily accessible than the intra-abdominal aorta in obese patients. Also, the descending thoracic aorta is often spared from severe atherosclerotic disease as compared with the intra-abdominal aorta.^11^ Finally, the right atrial appendage approach to IVC cannulation is more straightforward than gaining access to the IVC at the level of the aortic bifurcation. In our experience, it is likely these technical advantages that make DT-NRP a faster approach to cannulation versus IA-NRP. In terms of duration of NRP, the DT-NRP group was on NRP for longer than the IA-NRP group. This may reflect more stability on the pump that allows for longer duration without compromising the delivery of oxygen to the abdominal organs. It is also likely secondary to 1 confounding factor in some abdominal cases, which is the presence of a thoracic team performing rapid recovery of the lungs and/or heart. In these cases, volume loss in the chest during the cold dissection can impact stability on the pump, increase blood transfusion requirements, and require shortening perfusion time. Additionally, NRP duration was determined by the preferences and logistical needs of the surgical teams rather than a predefined physiologic endpoint, and longer perfusion times were generally possible in cases demonstrating greater hemodynamic stability on the pump.

The improved flow dynamics observed in the DT-NRP group provide a physiologic explanation for several of our findings. DT-NRP achieved higher peak and sustained flow rates compared with IA-NRP despite similar calculated target flows, indicating a technique-driven advantage. When scaled to target, DT-NRP consistently achieved or exceeded calculated flows, whereas IA-NRP frequently failed to meet calculated flows. These differences likely reflect improved venous drainage via the right atrium, reduced abdominal compression, and less volume loss. This enhanced hemodynamic profile may contribute to improved lactate clearance and reduced transfusion requirements, supporting DT-NRP as a more physiologically optimized perfusion strategy.

The current study also found that the median decrease in lactate was greater in the DT-NRP group and that transfusion requirements were lower in the DT-NRP group. Greater lactate clearance could be related to several factors. First, it could be indicative of better perfusion to the liver and kidneys, leading to quicker functional recovery, as evidenced by better clearance. Second, it could be reflective of less lactate accumulation or introduction into the system. IA-NRP perfuses only the area from the aortic bifurcation to the diaphragm. Deoxygenated, lactate-rich venous blood from the thoracic cavity and upper extremities is collected from the proximally vented aorta and returned into the circuit, which serves as an ongoing source of lactate introduction. Moreover, banked blood has been shown to have high concentrations of lactate, so the increased number of transfusions associated with IA-NRP could be contributing to additional lactate introduction into the system. Finally, the duration of NRP may confound interpretation of delta lactate, as longer time on NRP provides greater opportunity for lactate reduction. In terms of transfusion requirements, there are several reasons that IA-NRP may require more blood. First, as noted above, IA-NRP can be paired with thoracic rapid recovery, which often leads to more blood and volume loss through the chest during the removal of thoracic organs. This alone may be the reason for higher blood product requirements in IA-NRP cases and needs to be studied in a larger series that can compare IA-NRP with and without thoracic rapid recovery. Second, volume loss from dissection or around the cannulation sites might be more significant with the IA-NRP approach. While our study found differences in delta lactate and red blood cell transfusions, more research is needed to determine the drivers of these differences.

Finally, we found that both procedures had a decline in cannulation times over case numbers, followed by an increase after 50 cases. DT-NRP had a sharper decline and lower nadir, suggesting a quicker learning curve and an overall more efficient procedure. The upward slope at the end of the study period could be because of the introduction of new staff to the techniques or the willingness to take on more complex cases.

This study has several limitations. First, it is a single group of cardiac perfusionists who had 3 y of NRP experience before the initiation of the study, so the perfusion protocols for A-NRP were well established. Second, all cannulating surgeons were experienced in rapid recovery donation after circulatory death procedures and underwent A-NRP workshop training and proctoring before the initiation of the study. Therefore, the results of this study may not be generalizable to novice perfusion or surgical NRP teams. Third, this is a retrospective study, so the choice of technique was not randomized, and there could be selection bias in how cases were performed. Moreover, all thoracic rapid recovery cases required an IA-NRP approach. These are the most complicated NRP cases because of blood loss in the chest during thoracic organ recovery. This subset of cases may be the reason for higher blood product requirements, shorter pump durations, and lower lactate clearance in the IA-NRP group. Further research is needed to separate out IA-NRP cases with and without thoracic rapid recovery to determine if this is the case, but in this study, the number of cases was not sufficient to do so. Finally, the focus of this study was solely on procedural parameters that were collected from perfusion records. We did not have access to organ utilization data or recipient outcome data, both of which are essential for a comprehensive comparison of techniques and will be part of a future multicenter research collaboration. NRP services assessed in this study were provided to multiple organ procurement organizations, while decisions regarding donor selection and organ utilization were made independently by transplant centers. Selection bias may have influenced both organ recovery practices and utilization patterns.

In conclusion, we found the DT-NRP approach was associated with shorter cannulation time and lower blood product utilization. A steeper initial decline and lower nadir in the DT-NRP learning curve indicate this technique may have a quicker learning curve and be a more efficient approach to cannulation. DT-NRP cannulation is a technique that can be implemented by A-NRP programs with a quick learning curve, providing a versatile option for cannulation in these cases.

## Supplementary Material


